# IgG4-related hepatic inflammatory pseudotumor with sclerosing cholangitis: a case report and review of the literature

**DOI:** 10.4076/1757-1626-2-7029

**Published:** 2009-06-11

**Authors:** Itaru Naitoh, Takahiro Nakazawa, Hirotaka Ohara, Tomoaki Ando, Kazuki Hayashi, Hajime Tanaka, Fumihiro Okumura, Hitoshi Sano, Takashi Joh

**Affiliations:** 1 Department of Gastroenterology and Metabolism, Nagoya City University Graduate School of Medical Sciences1 Kawasumi, Mizuho-cho, Mizuho-ku Nagoya 467-8601Japan; 2 Department of Gastroenterology, Gifu Prefectural Tajimi Hospital5-161 Maehata-cho, Tajimi, 507-8522Japan

## Abstract

**Introduction:**

Inflammatory pseudotumor is rare benign mass composed of chronic inflammatory cell infiltration and proliferating fibrous tissue. Some cases of inflammatory pseudotumor show abundant infiltrating IgG4-positive plasma cells and obliterative phlebitis, which are the pathologic hallmarks of autoimmune pancreatitis.

**Case presentation:**

A 77-year-old Japanese man was admitted to our hospital because of epigastric pain. A solitary mass with delayed enhancement by dynamic computed tomography was present in the left hepatic lobe. Endoscopic retrograde cholangiography showed only segmental stenosis of the left intrahepatic bile duct. No abnormal findings were detected in the pancreas. The patient was clinically diagnosed as having intrahepatic cholangiocarcinoma and underwent surgery. Histological examination of the hepatic mass and bile duct wall showed abundant IgG4-positive plasma cell infiltration with obliterative phlebitis. The final diagnosis was IgG4-related hepatic inflammatory pseudotumor with sclerosing cholangitis. Delayed enhancement by computed tomography is a characteristic feature of IgG4-related inflammatory pseudotumor similar to that of autoimmune pancreatitis.

**Conclusion:**

IgG4-related hepatic inflammatory pseudotumor unassociated with autoimmune pancreatitis should be one of the entities considered for differential diagnosis of liver tumors. Delayed enhancement on computed tomography might be useful finding for diagnosing IgG4-related hepatic inflammatory pseudotumor.

## Introduction

Inflammatory pseudotumor (IPT) of the liver is a rare, benign lesion composed of chronic inflammatory cell infiltration and proliferating fibrous tissue. This disease is becoming an important entity to consider for differential diagnosis in patients with hepatic space-occupying lesions.

Recently, hepatic IPT cases associated with autoimmune pancreatitis (AIP) have been reported [1-3] since the concept of AIP was established. IgG4 is reportedly a useful marker for discriminating AIP from other pancreatic and biliary diseases [4]. AIP is often associated with systemic extrapancreatic lesions [5]. Therefore, the concept of IgG4-related autoimmune diseases has been proposed by Kamisawa et al., who have demonstrated a number of IgG4 antibody-stained plasma cells in a number of organs in the human body [6]. Some cases of IPT show infiltration of abundant IgG4-positive plasma cells and obliterative phlebitis, which are the pathologic hallmarks of AIP [7,8]. Hepatic inflammatory pseudotumor can be pathologically classified into two types, of which the lymphoplasmacytic type could belong to the so-called IgG4-related diseases [9].

We have previously reported that AIP is frequently associated with sclerosing cholangitis (SC) [10]. SC associated with AIP (SC with AIP) differs from primary sclerosing cholangitis (PSC) in terms of symptoms, associated diseases, level of IgG4 and clinical course. SC with AIP has a cholangiographic appearance similar to that of PSC [11]. It must be emphasized that the presence of pancreatic abnormalities is the most useful feature for distinguishing PSC from SC with AIP. However, some cases of SC showing no abnormal changes in the pancreas could be included in the same category of AIP.

## Case presentation

A 77-year-old Japanese man with a 30-year history of alcohol-drinking was admitted to our hospital because of epigastric pain. His laboratory data on admission showed an elevated level of γ-glutamyl transpeptidase (114 IU/l).

**Figure 1.**
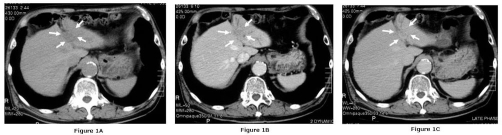
CT reveals a low-density mass (arrows) in the left lateral segment **(A)** with slight enhancement in the early phase **(B)** and further enhancement in the delayed phase **(C)**.

Computed tomography (CT) revealed a low-density mass in the left lateral segment. The mass was slightly enhanced in the early phase and further enhanced in the delayed phase (Figure 1A, 1B, & 1C). The mass and the hepatic segment 3 were not enhanced in CT during arterial portograpy.

**Figure 2.**
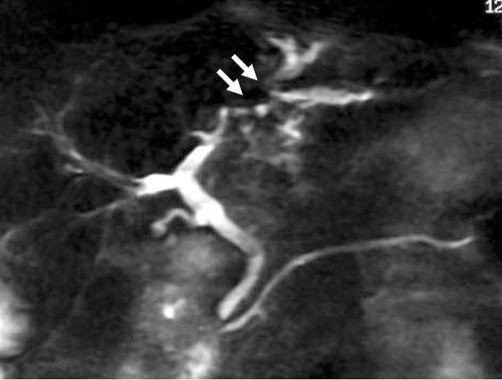
Magnetic resonance cholangiopancreatography reveals stricture of the intrahepatic bile ducts with prestenotic dilatation in the left lateral segment (arrows).

Magnetic resonance imaging (MRI) showed that the mass was slightly hypointense on T1-weighted images, and slightly hyperintense on T2-weighted images. Magnetic resonance cholangiopancreatography (MRCP) revealed stricture of the intrahepatic bile ducts with prestenotic dilatation in the left lateral segment (Figure 2). Endoscopic retrograde cholangiography (ERC) revealed bile duct stenosis in the left lateral lobe and prestenotic dilatation of the bile duct. Brush cytology specimens obtained from the stenotic portion of the bile duct showed no malignancy. Although no direct evidence of malignancy was obtained, intrahepatic cholangiocarcinoma was diagnosed on the basis of the imaging findings, and surgical left lobectomy was performed.

**Figure 3.**
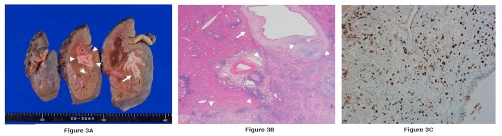
**(A)** Macroscopic examination shows a whitish nodular mass spreading along the hilar bile duct to B3. Arrowheads show the hepatic IPT and arrow shows the bile duct wall. **(B)** Histological appearance, with diffuse and severe lymphoplasmacytic infiltration (Hematoxylin & Eosin). Arrowheads show the hepatic inflammatory pseudotumor and arrow shows the bile duct wall. **(C)** Immunohistochemistry demonstrates abundant plasma cells positively stained by anti-IgG4 antibody.

Macroscopic examination of the resected left hepatic lobe revealed a whitish nodular mass measuring 4 x 3 cm spreading along the hilar bile duct to B3 (Figure 3A). The proximal bile duct was dilated by the mass.

Histological examination showed diffuse and severe lymphoplasmacytic infiltration (Figure 3B) and obliterative phlebitis. The bile duct was thickened and showed SC with diffuse fibrosis and prominent lymphoplasmacytic infiltration. Immunohistochemically, abundant plasma cells were positively stained by anti-IgG4 antibody (Figure 3C). Postoperative laboratory values were IgG4 231 mg/dl (normal value <134 mg/dl). The final diagnosis was IgG4-related hepatic IPT with SC.

## Discussion

Histopathologically, this case of hepatic IPT showed the characteristic features of plasma cell granuloma. Abundant plasma cells were positively stained with anti-IgG4 antibody, and obstructive phlebitis was evident. These findings were similar to those of AIP. This IPT protruded from the thick wall of the bile duct and was associated with SC. There was no evidence of AIP in the pancreas.

IPT can occur in various organs such as the lungs, liver, spleen and pancreas, and is a heterogeneous disease entity. Histologically, IPT has been subcategorized as plasma cell granuloma or inflammatory myofibroblastic tumor. IPTs of the plasma cell granuloma type show infiltration of numerous IgG4-positive plasma cells and obliterative phlebitis, which are the pathologic hallmarks of AIP [7,8].

The etiology of hepatic IPT remains elusive. Previous reports have indicated that cholangitis can be responsible for its development [12-14]. The 16 reported cases of hepatic IPT are pathologically classified into two types: the fibrohistiocytic type (10 cases) and the lymphoplasmacytic type (6 cases). The lymphoplasmacytic type could belong to the so-called IgG4-related diseases [9]. The present case was also the lymphoplasmacytic type and showed abundant infiltrating IgG4-positive plasma cells and obliterative phlebitis. Recently, more cases of hepatic IPT associated with AIP [1-3] have been reported, following recognition of the concept of AIP. The presence of pancreatic abnormalities is the most useful feature for diagnosis of SC with AIP. The present patient had no pancreatic enlargement or diffuse narrowing of the main pancreatic duct. Therefore, precise diagnosis of biliary stenosis was very difficult. Zen et al. proposed a concept of IgG4-related SC with and without hepatic IPT belonging to a spectrum of AIP [7], which was classified into six groups on the basis of association with/without SC and hepatic IPT. The present case was SC and hepatic IPT without AIP.

Autoimmune hepatitis (AIH) is one of the autoimmune disorders of the liver and histoligically characterized by a dense mononuclear and plasma cell infiltration of the portal areas. However, AIH usually do not form mass and abundant IgG4-positive plasma cells are not observed. These points were different from IgG4-related hepatic IPT.

Table 1 summarizes the eight cases of hepatic IPT that were proved to be IgG4-related [1-3,7]. All the patients were males aged between 48 and 79 years, with an average age of 62.6 years. Five of the cases were misdiagnosed as intrahepatic cholangiocarcinoma, and three cases were associated with AIP. Surgical treatment was unnecessary in two cases because they were definitively diagnosed by needle liver biopsy.

**Table 1.** Summary of reported eight and our lgG4-related hepatic IPT cases

case	Age (Y)	sex	Clinical Diagnosis	Solitary/multiple	Pancretic IPT	SC	Treatment
1	59	M	Liver cancer	solitary	−	+	Segmentectomy of the liver
2	79	M	ICC	solitary	head	+	Segmentectomy of the liver
3	56	M	ICC	solitary	−	+	Lobectomy of the liver
4	64	M	ICC	solitary	−	+	Lobectomy of the liver
5	67	M	Hepatic hilar cholangiocarcinoma	solitary	−	+	Lobectomy of the liver
6	59	M	Hepatic IPT+AIP	multiple	head	IHBD CBD (MRCP)	PSL
7	48	M	ICC+AIP	multiple	head	lowerCBD (ERCP)	Lobectomy of the liver+PSL
8	54	M	Hepatic IPT+AIP	solitary	head	lowerCBD (ERCP)	No treatment
our case	77	M	ICC	solitary	−	+	Lobectomy of the liver

ICC, Intrahepatic cholangiocarcinoma; AIP, Autoimmune pancrealitis; IPT, Inflammatory pseudotumor; PSL, Prednizolone.

case1-5 cited from reference 7, case6 from reference 1, case7 from reference 2, case8 from reference 3.

The present case was treated surgically under a suspected diagnosis of intrahepatic cholangiocarcinoma because of segmental bile duct stricture and portal obstruction. After surgery, we reassessed the imaging findings, and noted homogeneous delayed enhancement on dynamic CT, whereas MRI showed low intensity in the T1- and slightly high intensity in the T2-weighted images, similar to the CT and MRI features of AIP. We then evaluated the CT findings for five cases of IgG4-related IPT we had encountered (one spleen, one pancreas, two lung, one liver). All of these cases showed delayed enhancement. Three cases in which the lesions were small showed homogeneous enhancement, while two cases in which the lesions were large showed heterogeneous enhancement.

## Conclusion

IgG4-related hepatic inflammatory pseudotumor unassociated with autoimmune pancreatitis should be one of the entities considered for differential diagnosis of liver tumors. IgG4-related hepatic IPT with sclerosing cholangitis had the characteristic CT findings of the other IgG4-related IPT. Delayed enhancement on CT might be useful finding for diagnosing IgG4-related hepatic IPT. If the serum IgG4 level is elevated, liver tumor biopsy and IgG4 staining are recommended.

## List of abbreviations

AIH: Autoimmune hepatitis
AIP: Autoimmune pancreatitis
CT: Computed tomography
ERC: Endoscopic retrograde cholangiography
IPT: Inflammatory pseudotumor
MRCP: Magnetic resonance cholangiopancreatography
PSC: Primary sclerosing cholangitis
SC: sclerosing cholangitis
